# Posaconazole-Induced Adrenal Insufficiency in a Case of Chronic Myelomonocytic Leukemia

**DOI:** 10.1155/2018/2170484

**Published:** 2018-01-16

**Authors:** Ann Miller, Lauren K. Brooks, Silpa Poola-Kella, Rana Malek

**Affiliations:** ^1^Department of Medicine, University of Maryland Medical Center, Baltimore, MD, USA; ^2^Department of Medicine, Division of Endocrinology, Diabetes and Nutrition, University of Maryland Medical Center, Baltimore, MD, USA

## Abstract

**Introduction:**

Posaconazole is an azole used in treatment and prophylaxis of a broad spectrum of fungal infections. Antifungals such as ketoconazole have been shown to cause primary adrenal insufficiency (AI) as a result of direct inhibition on the steroidogenesis pathway. There is only one reported case of primary AI induced by posaconazole in a patient with mucormycosis. We report a case of posaconazole-related primary AI.

**Case:**

A 63-year-old man with chronic myelomonocytic leukemia was admitted for fatigue and intermittent nausea and vomiting. He had recently discontinued prophylactic posaconazole 300 mg daily. He was assessed for AI with a morning cortisol of 1.9 mcg/dL followed by a failed cosyntropin stimulation (CS) test. Adrenocorticotropic hormone (ACTH) level was 154.6 pg/mL with negative 21-hydroxylase antibodies. The patient's symptoms improved with initiation of hydrocortisone and fludrocortisone. One year after discontinuation of posaconazole, he underwent a repeat CS test which showed normal adrenal function with normal ACTH at 34.1 pg/mL.

**Conclusion:**

In this case, we demonstrate that prolonged use of posaconazole is associated with primary AI. As use of posaconazole increases, knowledge of the potential risk of AI is important and must be included in the differential diagnosis when these patients present with hypotension, hypoglycemia, and failure to thrive.

## 1. Introduction

Adrenal insufficiency occurs when there is inadequate secretion of cortisol from the adrenal glands due to either failure of the adrenal glands or other causes such as critical illness or pituitary adrenocorticotropic hormone (ACTH) deficiency. Primary adrenal insufficiency is rare and is caused by insufficient cortisol production from the adrenal glands. Lab abnormalities include very low cortisol and significantly elevated ACTH. This is in contrast to secondary adrenal insufficiency in which there is an abnormality at the level of the pituitary gland, resulting in insufficient secretion of ACTH to stimulate cortisol production. Patients will concomitantly have low or inappropriately normal ACTH levels and low cortisol levels. The cosyntropin test can help distinguish between primary and secondary adrenal insufficiency. A normal adrenal gland will secrete cortisol in response to the administration of cosyntropin and reach a peak of >500 nmol/L thirty minutes later. Primary adrenal insufficiency will result in little or no increase in cortisol production. Measurement of ACTH can further help delineate primary versus secondary adrenal insufficiency [1].

The clinical presentation of adrenal insufficiency depends on disease chronicity and the presence of physical stressors. Manifestations of adrenal crisis include shock, hypotension, fever, nausea, vomiting, abdominal pain, tachycardia, and death. Causes of primary adrenal insufficiency include autoimmune adrenalitis, infections such as tuberculosis, disseminated fungal infections, human immunodeficiency virus, hemorrhagic infarction, or metastatic cancerous infiltration of the adrenal glands, and drugs.

Ketoconazole and posaconazole are both members of the azole antifungal family. They are primarily used as antifungal agents. Azole antifungals work by inhibiting the cytochrome P450 dependent enzyme lanosterol 14 alpha-demethylase, an enzyme necessary for the conversion of lanosterol to ergosterol, a vital component of the cell membrane in fungi. This enzyme is not present in mammalian cells.

**Figure 1:**
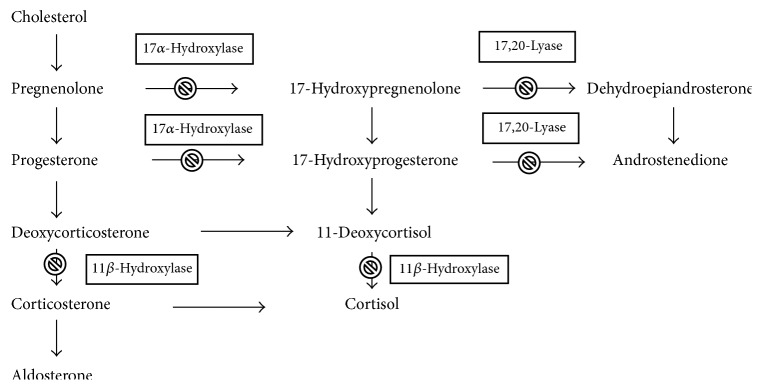
Diagram of the steroid synthesis pathway showing the steps where ketoconazole inhibits synthesis.

Ketoconazole is known to inhibit human steroidogenesis [2]. It impedes human steroid production by inhibiting human cytochrome P450 enzymes including the cholesterol side-chain cleavage complex 17,20-lyase, 11*β*-hydroxylase, and 17*α*-hydroxylase [3] (Figure 1). Due to its steroidogenesis inhibitory effects, ketoconazole can be used as a treatment for Cushing's syndrome. While its use has been shown to effectively reduce urinary free cortisol in patients with Cushing's syndrome, adrenal insufficiency is a known side effect of therapy [4, 5]. In 2013, the US Food and Drug Administration (FDA) limited the use of Nizoral (ketoconazole) oral tablets due to the side effects of hepatotoxicity and adrenal insufficiency [6].

Ketoconazole is an imidazole derivative, meaning that there are 2 nitrogen atoms in the azole ring. Triazoles are members of the azole class that have 3 nitrogen atoms in the azole ring. First-generation triazoles include fluconazole and itraconazole and second-generation triazoles include posaconazole and voriconazole. The most recent azole to be approved for use against invasive aspergillosis and invasive mucormycosis infections is isavuconazole. It was approved by the FDA in 2015.

Posaconazole has been FDA approved for the prophylaxis and treatment of refractory invasive fungal infections. During phase I, II, and III clinical trials, posaconazole was found to be safe and well tolerated. During phase I studies, the most common side effects included gastrointestinal symptoms, headache, dry mouth, somnolence, dizziness, and constipation. These side effects were mild and transient in nature [7]. Here we present a case in which posaconazole is theorized to be the cause of primary adrenal insufficiency.

## 2. Case

A 63-year-old African-American male with type 2 diabetes on metformin, rheumatoid arthritis not on oral corticosteroid therapy, chronic low back pain alleviated with intermittent epidural steroid injections, hepatitis C, and chronic myelomonocytic leukemia (CMML) who was on decitabine for chemotherapy presented to the hospital on 6/25/16 for hematemesis and poor oral intake. Prior to arrival, he had been feeling weak and his weakness acutely worsened in the two to three weeks before admission. His other symptoms included nausea and poor appetite. He had vomited at least twice. Two days prior to his admission, he was seen in his outpatient oncologist's office. He told his oncologist that he had not been feeling well and had been experiencing dehydration and fatigue. He said his symptoms started around the time the posaconazole 300 mg daily was started in April of 2016 for fungal prophylaxis. His oncologist held his posaconazole at that visit on 6/23/16. The only relevant steroid exposure was intermittent steroid injections into his spine for low back pain. His last injection had been in March of 2016. It was estimated that he had received a total of five steroid injections into his back prior to admission. The time interval between and the time course of injections are not specified. His full medication list included potassium supplementation, hydroxychloroquine, indomethacin, tramadol, docusate, acyclovir, levofloxacin, decitabine, allopurinol, gabapentin, aspirin, lisinopril, and metformin.

Blood pressure on admission was within normal limits at 125/60 mmHg. Labs on admission showed hypokalemia (3.1 mmol/L) and hypocalcemia (8.5 mg/dL) but normal sodium and glucose. To evaluate his adrenal function, the primary team sent a morning cortisol on 6/26/16 which resulted as 1.9 mcg/dL at 5:39 AM. To further evaluate his adrenal function, he underwent a cosyntropin stimulation test. On the morning of 6/27/16, his cortisol at 5:28 AM was 2.2 mcg/dL. He was then given one dose of cosyntropin 250 mcg IV at 8:00 AM with repeat cortisol measurements resulting in 3.7 mcg/dL at 30 minutes and 4.1 mcg/dL at 60 minutes. A CT scan of the abdomen and pelvis without contrast was done on 6/26/16 and showed bilateral thickened adrenal glands consistent with hyperplasia. During that admission, he was started on hydrocortisone 15 mg by mouth in the morning and 10 mg by mouth in the evening. ACTH measured on 6/27/16 was 154.6 pg/mL. After the ACTH results returned, he was started on fludrocortisone 0.1 mg by mouth daily as the providers concluded that the patient had primary adrenal insufficiency given his elevated ACTH and failed cosyntropin test. There was no renin value measured at the time of initial diagnosis.

He was followed up in the endocrine clinic one month later. During the office visit, it was documented that he had hyperpigmentation of the skin but the patient stated that this had been present for years. After starting hydrocortisone and fludrocortisone, his symptoms improved including increased appetite and resolution of headache and nausea. He was rehospitalized in September 2016 for a stem cell transplant for his CMML. 21-OH antibodies were tested during that admission and were negative (<0.1). On discharge, he was prescribed hydrocortisone 10 mg by mouth in the morning and 5 mg by mouth in the afternoon. He tolerated this regimen well. He re-presented to the endocrine clinic in July of 2017 (one year after discontinuation of posaconazole) for repeat cosyntropin testing. Cosyntropin 250 mcg IV was given after measurement of a baseline cortisol of 5 mcg/dL. One hour after the cosyntropin was given, it rose to 18.5 mcg/dL. Pretesting ACTH was 34.1 pg/mL (7–69 pg/mL) and renin was 0.176 ng/mL/hr (0.167–5.380 ng/mL/hr). Based on his improved cosyntropin testing (Table 1), he discontinued the hydrocortisone and fludrocortisone.

## 3. Discussion

This case report describes a patient presenting with nausea and vomiting in the setting of recently taking posaconazole who was found to have failed a cosyntropin test with simultaneous elevated ACTH suggesting posaconazole-induced primary adrenal insufficiency that resolved after discontinuation of the medication.

There are several case reports of azoles causing adrenal insufficiency. These generally have been in the setting of drug-drug interactions when azoles are taken concomitantly with steroids. With regard to the first-generation triazoles, there are three documented cases of fluconazole-induced adrenal insufficiency in the critically ill [8, 9], two case reports of fluconazole-induced adrenal insufficiency due to drug interactions leading to synergistic inhibition of the adrenal gland [10, 11], and only one documented case report of fluconazole-induced acute primary adrenal insufficiency when it was used as a prophylactic measure against fungal infections in a patient undergoing high dose cytotoxic chemotherapy [12]. What many of these cases share is the inhibition of CYP3A4 which results in supraphysiologic levels of corticosteroids (inhaled, intranasal, intra-articular, or oral) leading to iatrogenic Cushing's syndrome with resultant adrenal suppression. There are also case reports of itraconazole-induced central adrenal insufficiency, but in all cases, it was thought to be due to a drug interaction with inhaled corticosteroids [13, 14]. The etiology of itraconazole-induced adrenal insufficiency due to drug interactions with inhaled corticosteroids was further evaluated by Skov et al. in 2002 [15]. The researchers from Denmark evaluated adrenal function in patients with cystic fibrosis or chronic granulomatous disease who were treated for allergic bronchopulmonary aspergillosis with itraconazole either alone (*N* = 12) or with inhaled budesonide (*N* = 25). Eleven of 25 patients who were treated with combination itraconazole and inhaled budesonide did not respond to ACTH stimulation testing. These were the only patients who experienced any evidence of adrenal insufficiency. Of these patients, 8 were also found to have low plasma ACTH levels. These findings suggest that itraconazole decreases the CYP3A4 metabolism of budesonide causing increased systemic levels of the steroid leading to central adrenal insufficiency.

With regard to incidences of adrenal insufficiency in second-generation triazoles, there is only one case report of voriconazole induced central adrenal insufficiency, again with the thought being the adrenal insufficiency was due to a drug interaction with inhaled corticosteroids [16]. There is only one documented case of posaconazole-induced adrenal insufficiency [17]. The patient had a past medical history significant for type 1 diabetes and was hospitalized for diabetic ketoacidosis and developed rhino-orbital mucormycosis. The patient was treated with posaconazole, starting on day 10, and it was continued on discharge, day 55. She was noted to develop falling insulin requirements and progressive hypotension. Further work-up revealed a failed cosyntropin stimulation test and negative anti-adrenal antibody testing which led the authors to conclude that posaconazole was the cause of the patient's adrenal insufficiency. We present a similar case where posaconazole was thought to be the cause of adrenal insufficiency but we were able to follow the ACTH and repeat the cosyntropin test and show resolution of the adrenal function after discontinuation of the posaconazole. There have been no case reports of isavuconazole induced adrenal insufficiency.

We argue that our patient experienced primary adrenal insufficiency given his lab findings of elevated ACTH and failed cosyntropin testing. Further information to support this diagnosis is the bilateral adrenal hyperplasia found on his abdominal CT scan. The pathophysiology of our patient's adrenal hyperplasia could be similar to that of congenital adrenal hyperplasia where cortisol synthesis is also impaired, although in most cases due to the deficiency of 21-hydroxylase. The decreased cortisol production in both cases leads to increased ACTH secretion causing hyperplasia of the adrenal glands [18]. The difference in the two clinical scenarios is the mechanism of inability for cortisol production.

Our patient's potassium and sodium levels were not suggestive of primary adrenal insufficiency as it usually presents with hyponatremia and hyperkalemia. However, these lab abnormalities could be explained by his vomiting and infusions. The vomiting caused hypokalemia due to gastrointestinal losses and his sodium may have been normal due to recent boluses of normal saline outpatient, the most recent on 6/24/16, the day prior to admission.

With respect to other etiologies of the patient's primary adrenal insufficiency, autoimmune disease was ruled out by his negative 21-OH antibodies and adrenal hemorrhage was not present on his imaging. Finally, his facial hyperpigmentation predated his acute adrenal insufficiency and was unlikely to be related to his short standing elevation of ACTH.

While we argue that this patient experienced primary adrenal insufficiency from posaconazole inhibition of steroidogenesis, there are weaknesses in our case that we must consider. Most importantly, the patient had been receiving corticosteroid injections for his back pain throughout this time, the timing of which is not well documented. Steroids can cause adrenal insufficiency due to their negative feedback on corticotropin releasing hormone and ACTH. This leads to adrenal atrophy and decreased cortisol secretion from the adrenal gland. However, his CT results showing adrenal hyperplasia and his elevated ACTH argue strongly for primary adrenal insufficiency.

## 4. Conclusion

Here we present a case of primary adrenal insufficiency attributed to long-term posaconazole use. As posaconazole use for the prophylaxis against and treatment of invasive fungal infections increases, clinicians should be aware of this possible long-term side effect. Clinicians should caution against its use in patients at risk of developing adrenal insufficiency. Clinical signs or symptoms of adrenal insufficiency, such as weight loss, hypotension, or hypoglycemia, may be clues suggesting the development of this side effect. Awareness of this side effect may also be warranted with the newest approved azole, isavuconazole.

## Figures and Tables

**Table 1:** Comparison of labs just after discontinuation of posaconazole and a year later.

Labs	2016	2017
ACTH (7–69 pg/mL)	154.6 (7–69 pg/mL)	34.1 (7–69 pg/mL)
Cortisol, morning baseline (4.4–22.7 mcg/dL)	2.2	5 mcg/dL
Cortisol, 30 minutes after cosyntropin (mcg/dL)	3.7	n/a
Cortisol, 60 minutes after cosyntropin (mcg/dL)	4.1	18.5 mcg/dL
